# Abnormal methylation characteristics predict chemoresistance and poor prognosis in advanced high-grade serous ovarian cancer

**DOI:** 10.1186/s13148-021-01133-2

**Published:** 2021-07-21

**Authors:** Li-yuan Feng, Bing-bing Yan, Yong-zhi Huang, Li Li

**Affiliations:** Department of Gynecology and Oncology, Guangxi Medical University Cancer Hospital and Key Laboratory of Early Prevention and Treatment for Regional High Frequency Tumor, Ministry of Education, 71 Hedi Road, Nanning, 530021 Guangxi People’s Republic of China

**Keywords:** Ovarian cancer, Chemotherapy resistance, DNA methylation, 450 K Infinium Methylation BeadChip, Prognosis

## Abstract

**Background:**

Primary or acquired chemoresistance is a key link in the high mortality rate of ovarian cancer. There is no reliable method to predict chemoresistance in ovarian cancer. We hypothesized that specific methylation characteristics could distinguish chemoresistant and chemosensitive ovarian cancer patients.

**Methods:**

In this study, we used 450 K Infinium Methylation BeadChip to detect the different methylation CpGs between ovarian cancer patients. The differential methylation genes were analyzed by GO and KEGG Pathway bioinformatics analysis. The candidate CpGs were confirmed by pyrosequencing. The expression of abnormal methylation gene was identified by QRT-PCR and IHC. ROC analysis confirmed the ability to predict chemotherapy outcomes. Prognosis was evaluated using Kaplan–Meier.

**Results:**

In advanced high-grade serous ovarian cancer, 8 CpGs (ITGB6:cg21105318, cg07896068, cg18437633; NCALD: cg27637873, cg26782361, cg16265707; LAMA3: cg20937934, cg13270625) remained hypermethylated in chemoresistant patients. The sensitivity, specificity and AUC of 8 CpGs (ITGB6:cg21105318, cg07896068, cg18437633; NCALD: cg27637873, cg26782361, cg16265707; LAMA3: cg20937934, cg13270625) methylation to predict chemotherapy sensitivity were 63.60–97.00%, 46.40–89.30% and 0.774–0.846. PFS of 6 candidate genes (ITGB6:cg21105318, cg07896068; NCALD: cg27637873, cg26782361, cg16265707; LAMA3: cg20937934) hypermethylation patients was significantly shorter. The expression of NCALD and LAMA3 in chemoresistant patients was lower than that of chemosensitive patients. Spearman analysis showed that NCALD and LAMA3 methylations were negatively correlated with their expression.

**Conclusions:**

As a new biomarker of chemotherapy sensitivity, hypermethylation of NCALD and LAMA3 is associated with poor PFS in advanced high-grade serous ovarian cancer. In the future, further research on NCALD and LAMA3 will be needed to provide guidance for clinical stratification of demethylation therapy.

**Supplementary Information:**

The online version contains supplementary material available at 10.1186/s13148-021-01133-2.

## Background

Ovarian cancer is the most lethal cancer of the female reproductive system. In 2019, 22,530 new cases and 13,980 death occurred in the USA [1]. For the first diagnosis of ovarian cancer patients, the standard treatment is the optimal surgical cytoreduction combined with platinum-based chemotherapy [2]. In the past decade, the survival rate of ovarian cancer has changed only a little, and the 5-year survival rate has remained below 30% [3, 4]. Primary or acquired chemoresistance is a key link in the high mortality rate of ovarian cancer [5, 6]. If the patient’s sensitivity to traditional chemotherapy can be assessed before treatment, doctors can guide chemoresistant patients to undergo clinical trials to seek survival opportunities. Therefore, it is necessary to use robust and sensitive biomarkers to predict chemotherapy outcome in ovarian cancer patients.

At present, there is no reliable method to predict chemoresistance in ovarian cancer. DNA methylation as a biomarker has the following advantages: chemical stability, quantitative detection, chemoresistance-related methylation changes usually occur before the start of chemoresistance, noninvasive detection (can be detected in the patient's body fluids) [7]. With the update of DNA methylation detection platform and technology, more and more genes involved in ovarian cancer chemoresistance have been reported. Except BRCA1, DNA damage repair pathway-related genes, PTEN, RASSF1, MDR1 and FANCF gene hypermethylation were positively correlated with chemotherapy sensitivity. In recent years, new DNA methylation studies related to chemotherapy resistance of ovarian cancer include at least MLH1 [8], SERPINE1 [9], TRIB2 [10], KLF4 [11], FZD10 [12], ZNF671 [13], ABCB1 [14], hMSH2 [15] and other genes. This shows that the regulatory mechanism of DNA methylation in ovarian cancer chemotherapy resistance is complex and diverse. A variety of methylated genes interact with each other, which together leads to chemotherapy resistance in ovarian cancer.

Relative to genetic mutations, DNA methylation can be reversed. Demethylation drugs can reverse abnormal methylation, improve the sensitivity of ovarian cancer patients to chemotherapy drugs, improve efficacy and prolong survival [16–18]. Currently, there are few results on genome-wide methylation in chemoresistant ovarian cancer patients. Here, we used 450 K Infinium Methylation BeadChip to study the genome-wide methylation characteristics of chemotherapy resistance in ovarian cancer.

## Materials and methods

### Patients

We collected the initial surgical samples of ovarian cancer patients (only carcinomas and not borderline tumors) from the Guangxi Medical University Cancer Hospital. A total of 108 frozen samples (epithelial ovarian cancer) were used for 450 K Infinium Methylation BeadChip, pyrosequencing and QRT-PCR. 132 paraffin samples (advanced high-grade serous ovarian carcinoma) were used for immunohistochemistry. All patients had complete chemotherapy outcome records and postoperative pathological diagnosis. All patients provided written informed consent and were approved by the institutional review committee of Guangxi Medical University Cancer Hospital.

### Follow-up

OS (overall survival) was defined as the time from the diagnosis to the death from ovarian cancer, and the survival data of the last follow-up survivors were recorded as censored data. PFS (progression-free survival) was defined as the time from initial treatment to tumor progression. Response to treatment was evaluated using the Response Evaluation Criteria In Solid Tumors (RECIST 1.1 criteria) [19]. Platinum-resistant and refractory ovarian cancer was defined as those whose disease had progressed during first-line platinum-based chemotherapy or relapsed within 6 months after the last platinum treatment [20]. Platinum-sensitive ovarian cancer was defined as relapse more than 6 months after platinum-based chemotherapy [21].

### Study design

In this study, we included two stages to identify and validate chemoresistance-related CpGs in ovarian cancer. In the discovery stage, 450 K Infinium Methylation BeadChip was used for screening, and enrichment analysis was used to select biologically meaningful CpGs. The relationship between abnormal methylation and chemotherapy resistance and prognosis was analyzed. In the verification stage, candidate CpGs were verified by pyrosequencing. To clarify whether differential CpGs play a biological function, we used QRT-PCR and immunohistochemistry to detect gene expression.

### 450K Infinium Methylation BeadChip

Qualified DNA was extracted from 108 samples. DNA was modified by Epitect bisulfite kit (Qiagen, Cat. No. 59110, Germany) and analyzed by 450 K Infinium Methylation BeadChip (Illumina, San Diego, CA, USA) in Shanghai Jingneng company. The methylation level was scored with standardized beta score values ranging from 0 (unmethylated) to 1 (fully methylated). Limma package (R) calculates differential methylation sites between chemotherapy-resistant and chemotherapy-sensitive patients (*P* ≤ 0.01, |Diff Beta Score| ≥ 0.1). Go (http://geneontology.org/) and KEGG (http://www.genome.jp/kegg/) databases were used to enrich the differential methylation genes. The candidate CpGs were selected according to the Diff Beta Score value and biological function.

### Literature search strategy

Gene search strategies were based on Yan's article [22]. Used ‘ovarian cancer’ or ‘ovarian carcinoma,’ ‘DNA methylation’ or ‘methylation,’ ‘resistant’ or ‘resistance’ or ‘chemoresistance’ as keywords, we screened methylated genes associated with the regulation of drug resistance in ovarian cancer from an advanced search in the PubMed database (http://www.ncbi.nlm.nih.gov/pubmed/). The search date was updated to May 4, 2021.

### Pyrosequencing

We used pyrosequencing to quantitatively determine the methylation level of candidate CpGs. Pyrosequencing primers were designed using PyroMark Assay Design 2.0 software. The primer sequences are shown in Additional file 1: Table S1. Bisulfite modified DNA amplified by PyroMark PCR Kit (Qiagen, Cat. No. 978703, Germany). The reaction steps were as follows: polymerase activation (95°, 3 min), 40 cycles of denaturation (94°, 30 s), annealing (52°, 30 s), extension (72°, 1 min) and final extension 72°, 7 min. The PCR products were qualitatively and quantitatively analyzed by 1% agarose gel electrophoresis.

### QRT-PCR

RNA extraction kit (Thermo Scientific, Cat. No. K0731, USA) for RNA extraction. Reverse transcription kit (Thermo Scientific, Cat. No. K1622, USA) for reverse transcription of cDNA. The polymerase chain reaction was performed used a fluorescent quantitative PCR kit (Taraka, Cat. No. DRR820A, Japan). The primer sequences are shown in Additional file 1: Table S2. The 2^−Δ*Ct*^ method calculated the relative expression levels of candidate genes. GAPDH was used as an internal control, and all experiments were repeated three times.

### Immunohistochemistry

NCALD, ITGB6 and LAMA3 concentration were 1:400 (Abcam, Cat. No.ab155161, UK), 1:20 (Abcam, Cat. No. ab197672, UK) and 1:50 (Abcam, Cat. No. ab217213, UK). NCALD, ITGB6, LAMA3 were mainly expressed in the cytoplasm, and a small amount was expressed in the cell membrane. Two pathologists read the pathological sections independently. The score criteria were as follows: Positive cell ratio of < 1, 1–25%, 25–50%, 50–75% and 75–100% were assigned 0, 1, 2, 3, 4 points, respectively. Stain intensity of no coloring, light yellow, yellow, brown was assigned 0, 1, 2, 3 points, respectively. The product of positive cell ratio and stain intensity was stain index. Stain index ≤ 6 points were classified as a low expression, while > 6 points were classified as a high expression [23].

### Statistical analysis

Except for 450 K Infinium Methylation BeadChip, the rest of the statistical analysis was performed using SPSS 17.0. ROC analysis confirmed the ability to predict chemotherapy outcomes. The cutoff point corresponding to the maximum Youden's index was the cutoff value. According to the cutoff value, patients with ovarian cancer were divided into hypermethylation and hypomethylation. *T* test (measurement data) and chi-square test (categorical data) were used for comparison between the two groups. The association between CpGs and prognosis was assessed by the Kaplan–Meier plotter. Spearman analyzed the relationship between two variables. *P* < 0.05 was considered statistically significant.

## Results

### Study population

The median age of patients was 50.98 ± 10.40 years old. The clinical characteristics are shown in Table 1. Among the frozen samples, 85 patients had FIGO stage III–IV and 23 patients had FIGO stage I–II. There were 91 patients with serous type, 14 patients with mucinous type and 3 patients with endometrioid histology types. There were 74 patients with high-grade serous carcinoma and 17 patients with low-grade serous carcinoma. There were 11 patients with grade 3 and 6 patients with grade 1–2 in mucinous carcinoma and endometrioid carcinoma. According to the postoperative chemotherapy scheme, there were 57 patients with TP (paclitaxel plus cisplatin) and 51 patients with TC (paclitaxel plus carboplatin). CpGs were verified in FIGO stage III–IV high-grade serous ovarian cancer, so the corresponding gene expression detection was carried out in advanced high-grade serous paraffin samples.

**Table 1:** Clinical characteristics of patients

Clinical characteristics	Frozen samples (N = 108)	Paraffin samples (N = 132)	All (N = 240)
Age	48.12 ± 11.47	53.32 ± 8.79	50.98 ± 10.40
*FIGO stage*			
I–II	23	–	23
III–IV	85	132	217
*Grade (serous)*			
3	74	132	206
1	17	–	17
*Grade (mucinous and endometrioid)*			
3	11	–	11
2	1	–	1
1	5	–	5
*Histology types*			
Serous	91	132	223
Mucinous	14	–	14
Endometrioid	3	–	3
*Surgical debulking*			
Optimal	76	96	172
Suboptimal	32	36	68
*Chemotherapy outcome*			
Chemoresistant	55	56	111
Chemosensitive	53	76	129
*Postoperative chemotherapy scheme*			
TP (paclitaxel plus cisplatin)	57	58	115
TC (paclitaxel plus carboplatin)	51	74	125

### Genome-wide DNA methylation between chemoresistant patients and chemosensitive patients in epithelial ovarian cancer

**Fig. 1:**
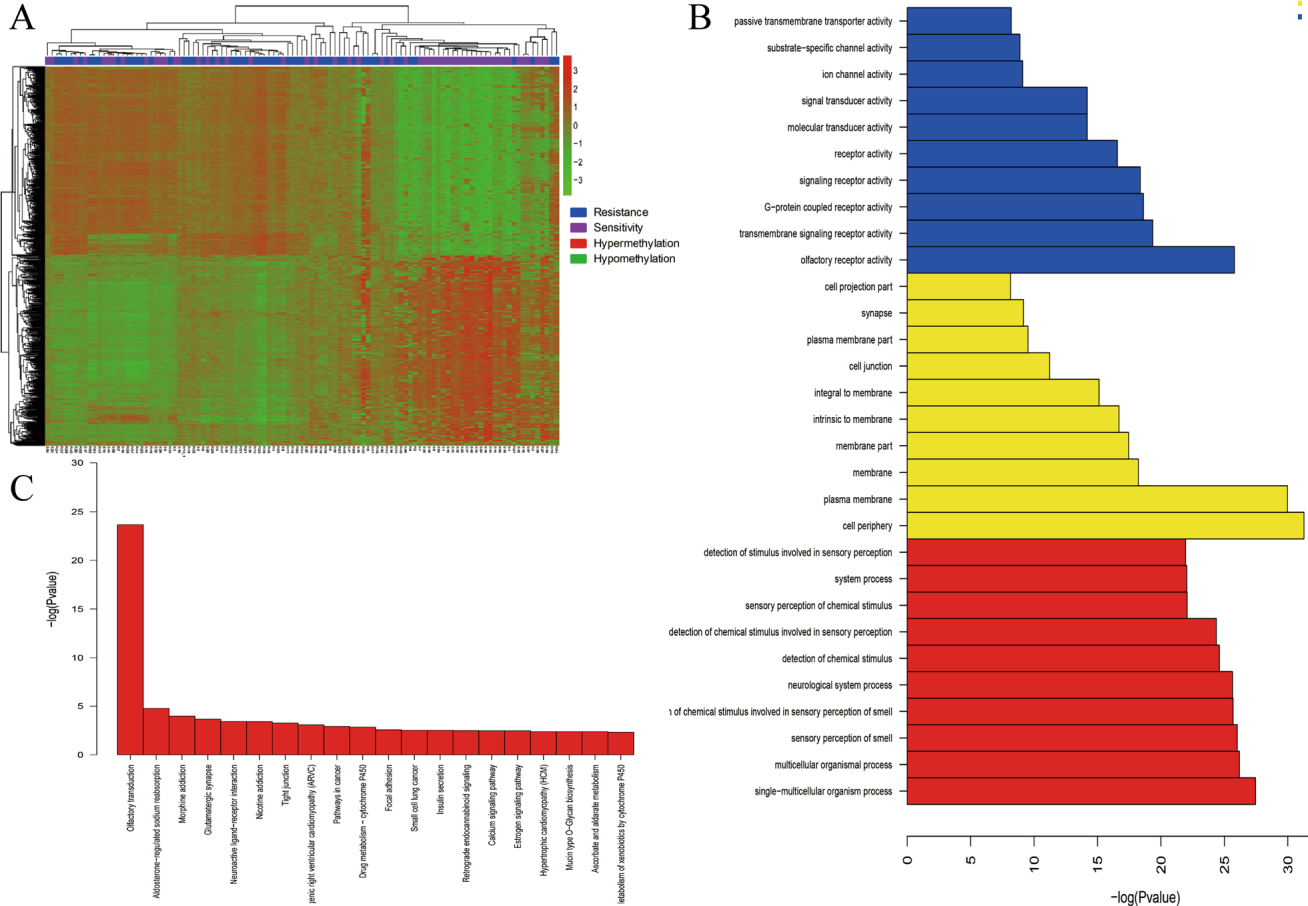
Genome-wide DNA methylation in epithelial ovarian cancer: **a** heat map of differential methylated genes. **b** The biological process of differential methylation genes (GO). **c** The signal pathways of differential methylation genes (KEGG)

The 450 K Infinium Methylation BeadChip contains 450,000 CpGs, covering 96% of CpG islands. Almost all the methylation genes annotated by NCBI are covered. Since most functionally related DNA methylation occurs on the CpG island of gene promoters, we prefer to select candidate CpG in the promoter region. 7263 CpGs showed significant differences between chemoresistant and chemosensitive epithelial ovarian cancer patients (55 chemoresistant and 53 chemosensitive patients), corresponding to 2654 genes. See Fig. 1a. Compared with chemosensitive patients, there are 6051 hypermethylated CpG loci (corresponding to 2162 genes) and 1212 hypomethylated CpG loci (corresponding to 452 genes) in chemoresistant patients. The difference CpGs in promoter region corresponds to 1058 genes, and the difference CpGs in the 5′UTR region corresponds to 305 genes. The signal pathways of differential methylation genes enriched in KEGG include drug metabolism-cytochrome P450, focal adhesion, calcium signaling pathway, PI3K-Akt signaling pathway and ErbB signaling pathway. Similarly, the biological process of GO enrichment includes the multicellular organismal process, cell adhesion, cell migration and calcium ion binding. See Fig. 1b, c and Additional file 1: Tables S3–S4.

We systematically searched the literature in the PubMed database and obtained 53 methylated genes related to chemotherapy resistance in ovarian cancer. Among them, 22 methylation genes related to ovarian resistance reported in the literature are enriched in our Methylation BeadChip results. The difference of BRCA1, CD133, ASS1, ABCG2, TGFBI, RGS10, UCHL1, CLDN4, HOXA10, DOK2, AGR2 and OXCT1 gene in our 450 K Infinium Methylation BeadChip is consistent with that reported in the literature. The difference of DNAJC15, RASSF1, HOXA9 and SFRP5 gene in our 450 K Infinium Methylation BeadChip is contrary to that reported in the literature. There is no significant difference in MLH1, FBXO32, PTEN, MAL, TUBB3, FANCF gene in our Methylation BeadChip, see Table 2 [9–12, 15, 24–65]. Because there are few studies on hypomethylation, we prefer to study hypermethylation genes in ovarian cancer chemoresistance. Based on the Diff Beta Score value, CpG region, KEGG and GO analysis, we selected 9 candidate CpGs with fewer reports in the current research literature, corresponding to 4 methylated genes (ITGB6:cg21105318, cg07896068, cg18437633; NCALD: cg27637873, cg26782361, cg16265707; PIK3R3: cg27584146; LAMA3: cg20937934, cg13270625). Diff Beta Score is given in Table 3.

**Table 2:** The methylated genes and ovarian cancer multidrug resistance in the literature

Author	Year	*N*	Population	Gene	Tissue	Methods	Cell	Methylation level in chemoresistant tissue/cell	Expression	Drugs	References
Strathdee	2005	41	UK	MCJ	Advanced EOC	Bisulfite sequencing	–	Hypermethylation	Downregulation	Cisplatin	[24]
Lee	2009	61	UK	MAL	SOC	Bisulfite sequencing	–	Hypomethylation	Upregulation	Platinum	[25]
Watanabe/Gifford	2007/2004	36/138	Japan/UK	hMLH1	Advanced EOC	MSP	–	Hypermethylation	Downregulation	Platinum	[26, 27]
Wang	2010	–	–	BRCA1	–	Bisulfite sequencing	COC1/COC1CisR	Hypomethylation	Upregulation	Cisplatin	[28]
Chou	2010	96	China	FBXO32	OC	MSP	–	Hypermethylation	Downregulation	Cisplatin	[29]
Nicholson	2009	54	UK	ASS1	EOC	Bisulfite sequencing	–	Hypermethylation	Downregulation	Cisplatin	[63]
GAO	2019	–	–	RASSF1A	–	MSP	A2780/A2780CisR	Hypermethylation	Downregulation	Cisplatin	[31]
GAO	2019	–	–	MDR1	–	MSP	A2780/A2780CisR	Hypomethylation	Upregulation	Cisplatin	[31]
Dai	2012	–	–	PTEN	–	Bisulfite sequencing	OVCAR3	Hypermethylation	Downregulation	Taxol	[32]
Li	2009	–	–	DR4	–	MSP	2008, Hey, NMP-1, OVCAR3, SKOV3, A2780	Hypermethylation	Downregulation	Platinum	[33]
Bram	2009	–	–	ABCG2	–	COBRA	IGROV1	Hypomethylation	Upregulation	Platinum	[34]
Chiang	2013	136	China	BLU	Advanced HGSOC	MSP	SKOV3, OVCAR3	Hypermethylation	Downregulation	Platinum	[35]
Wang	2012	40	China	TGFBI	EOC	MSP	SKOV3/SKOV3CisR, SKOV3/TR, A2780/TR	Hypermethylation	Downregulation	Cisplatin and palitaxel	[36]
Ali	2013	–	–	RGS10	–	Bisulfite sequencing	A2780/A2780CisR, CAOV3, SKOV3	Hypermethylation	Downregulation	Cisplatin	[37]
Okochi-Takada	2006	17	Japan	UCHL1	OC	MSP	13 ovarian cancer cell lines	Hypermethylation	Downregulation	Cisplatin	[38]
Staub	2007	–	–	HSulf-1	–	MSP	SKOV3, OV207	Hypermethylation	Downregulation	Platinum	[39]
Su	2010	126	China	SFRP5	EOC	MSP	–	Hypermethylation	Downregulation	Cisplatin	[40]
IZUTSU	2008	66	Japan	TUBB3	OC	Bisulfite sequencing	OVCAR-3, JHOC-8	Hypomethylation	Upregulation	Paclitaxel	[41]
Pattamadilok	2008	59	Thailand	LINE-1	EOC	COBRA	–	Hypomethylation	Unknown	Platinum	[42]
Jiang	2014	–	–	HOXA10	–	MSP	SKOV3, HEY	Hypomethylation	Upregulation	Platinum	[43]
Matei	2012	17	America	HOXA9, HOXA11	OC	Genome-wide methylation study	–	Hypermethylation	Unknown	Platinum	[44]
Taniguchi	2003	19	America	FANCF	OC	MSP	–	Hypomethylation	Upregulation	Cisplatin	[45]
YANG	2018	–	–	OXCT1	–	Genome-wide methylation study	8 ovarian cancer cell lines	Hypermethylation	Downregulation	Cisplatin	[46]
Zhao	2021	483	GEO and TCGA	AGR2, HSPA2, ACAT2	SOC	Genome-wide methylation study	–	Hypermethylation	Unknown	Platinum	[47]
Pan	2017	–	–	SERPINE1	–	MassArray EpiTYPER	A2780CP	Hypomethylation	Upregulation	Carboplatin	[9]
Zeller	2012	–	–	ARMCX2, COL1A1, MDK, MEST	–	Genome-wide methylation study	A2780CisR	Hypermethylation	Downregulation	Cisplatin	[48]
Ha	2018	–	–	NAGA	–	Genome-wide methylation study	11 ovarian cancer cell lines	Hypermethylation	Downregulation	Cisplatin	[49]
Visco	2021	16	America	CLDN1	Advanced SOC	Genome-wide methylation study	–	Hypomethylation	Upregulation	Cisplatin	[50]
Bonito	2016	61	UK	MSX1	HGSOC	Genome-wide methylation study	–	Hypomethylation	Upregulation	Platinum	[51]
Tomar	2016	–	–	CSK	HGSOC PDXs	Genome-wide methylation study	–	Hypermethylation	Downregulation	Platinum	[52]
Fang	2018	120	USA	DOK2	OC	Genome-wide methylation study	OVCAR3, SKOV3	Hypermethylation	Downregulation	Platinum	[53]
Yu	2011	–	–	PTK6, PRKCE, BCL2L1	–	Genome-wide methylation study	A2780CisR	Hypomethylation	Upregulation	Cisplatin	[54]
Teschendorff	2015	134	UK	HOTAIR	OC	Genome-wide methylation study	–	Hypomethylation	Upregulation	Platinum	[55]
Tian	2019	16	China	hMSH2	EOC	Bisulfite sequencing	–	Hypermethylation	Downregulation	Platinum	[15]
Syed	2011	52	Germany	Plk2	EOC	MSP	A2780CisR, SKOV3CisR	Hypermethylation	Downregulation	Cisplatin	[56]
Leon	2016	–	–	TMEM88	OC xenografts	Genome-wide methylation study	–	Hypomethylation	Upregulation	Platinum	[57]
Mase	2019	78	Japan/China	ZNF671	HGSOC	Genome-wide methylation study	JHOS2/4, OVCAR3	Hypermethylation	Downregulation	Platinum	[58]
Baba	2009	–	–	CD133/PROM1	–	MSP	OVCAR8/432, A2780, PEO1	Hypomethylation	Upregulation	Platinum	[59]
Shang/Litkouhi	2013/2007	–	–	CLDN4	–	MSP	2008	Hypermethylation	Downregulation	Cisplatin	[60, 61]
Witham	2008	–	–	DNAJC15	–	Bisulfite sequencing	OVCAR3/4/5/8, SKOV3	Hypermethylation	Downregulation	Platinum	[62]
Kritsch	2017	–	–	TRIB2	–	Genome-wide methylation study	A2780CisR, SKOV3CisR	Hypermethylation	Downregulation	Cisplatin	[10]
Lund	2017	–	–	KLF4	–	Genome-wide methylation study	M019iCisR, OC002CisR	Hypermethylation	Downregulation	Cisplatin	[11]
Tomar	2017	45	Netherlands	FZD10	HGSOC	Genome-wide methylation study	10 ovarian cancer cell lines	Hypomethylation	Upregulation	Platinum	[12]
Vaclavikova	2019	61	Czech Republic	ABCB1	EOC	Bisulfite sequencing	–	Hypomethylation	Upregulation	Platinum	[63]
Yao/Duan	2004/1999	–	–	TRAG-3/CSAG2	–	COBRA	SKOV3	Hypomethylation	Upregulation	Taxol	[30, 65]

*EOC* epithelial ovarian cancer, *SOC* serous ovarian cancer, *HGSOC* high-grade serous ovarian cancer, *MSP* methylation-specific polymerase chain reaction, *COBRA* combined bisulfite restriction analysis, *CisR* cisplatin resistant, *TR* palitaxel resistant, *CP* carboplatin resistant

**Table 3:** Hypermethylated genes in chemoresistant ovarian cancer patients (epithelial ovarian cancer)

Genes	Gene description	Diff beta score	*P* value
NCALD	Neurocalcin delta	0.202116802	2.16E−08
SLC1A6	Solute carrier family 1	0.169488766	3.19E−08
RXRG	Retinoid X receptor, gamma	0.165189407	6.28E−06
ITGB6	Integrin, beta 6	0.162725093	1.07E−05
DLG2	Disks, large homolog 2 (Drosophila)	0.161362933	1.22E−03
CTBP2	C-terminal binding protein 2	0.156564368	4.62E−06
LAMA3	Laminin, alpha 3	0.15511936	1.84E−07
OR1E2	Olfactory receptor, family 1, subfamily E, member 2	0.151817648	8.27E−08
OR5T3	Olfactory receptor, family 5, subfamily T, member 3	0.151025609	1.57E−07
OR4B1	Olfactory receptor, family 4, subfamily B, member 1	0.150708678	4.06E−08
EPB41L1	Erythrocyte membrane protein band 4.1-like 1	0.149492829	3.13E−05
OR8H3	Olfactory receptor, family 8, subfamily H, member 3	0.149428771	8.58E−08
GABRA6	Gamma-aminobutyric acid (GABA) A receptor, alpha 6	0.14743259	1.24E−07
OR10A5	Olfactory receptor, family 10, subfamily A, member 5	0.146965271	2.03E−06
RIMS1	Regulating synaptic membrane exocytosis 1	0.146489176	6.40E−07
GCNT3	Glucosaminyl (*N*-acetyl) transferase 3, mucin type	0.144933199	7.31E−06
MYH4	Myosin, heavy chain 4, skeletal muscle	0.14130487	1.29E−06
PIK3R3	Phosphoinositide-3-kinase, regulatory subunit 3 (gamma)	0.13020729	1.13E−03

### Genome-wide DNA methylation between chemoresistant patients and chemosensitive patients in advanced high-grade serous ovarian cancer

High-grade serous ovarian cancer (HGSOC) is the most common ovarian cancer subtype and accounts for 80% of the deaths caused by the disease. Advanced (FIGO stage III and IV) HGSOC is one of the hardest human malignancies to treat. We performed genome-wide methylation analysis in advanced high-grade serous ovarian cancer (33 chemoresistant and 28 chemosensitive patients). In advanced high-grade serous ovarian cancer, 3446 CpGs showed significant differences between chemoresistant and chemosensitive patients, corresponding to 855 genes. Compared with chemosensitive patients, there are 2707 hypermethylated CpGs (corresponding to 1611 genes) and 739 hypomethylated CpGs (corresponding to 344 genes) in advanced high-grade serous chemoresistant patients. In advanced high-grade serous ovarian cancer, 8 CpGs remained hypermethylated in chemoresistant patients (ITGB6:cg21105318, cg07896068, cg18437633; NCALD: cg27637873, cg26782361, cg16265707; LAMA3: cg20937934, cg13270625).The difference between the 6 CpGs (ITGB6:cg21105318, cg07896068, cg18437633; NCALD: cg27637873, cg26782361, cg16265707) in chemoresistant patients and sensitive patients is more than 0.2.

### The ability of candidate CpGs to predict chemotherapy sensitivity in advanced high-grade serous ovarian cancer

**Fig. 2:**
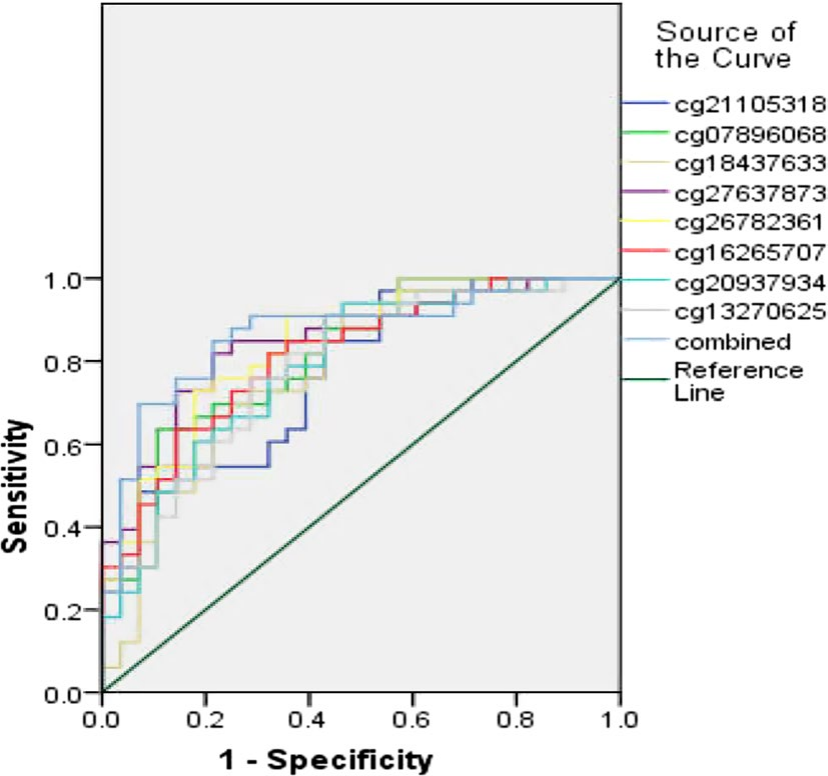
ROC curve of candidate CpGs to predict chemotherapy sensitivity in advanced HGSOC

There is a need to develop and validate biomarkers for chemotherapy response and survival in advanced high-grade serous ovarian cancer (*N* = 61). The sensitivity, specificity and AUC of 8 candidate CpGs methylation to predict chemotherapy sensitivity were 63.60–97.00%, 46.40–89.30% and 0.774–0.846. In SPSS, 8 CpGs were included in binary logistic regression to produce a predicted value. ROC analysis was performed on the predicted value. The sensitivity, specificity and AUC of 8 candidate CpGs methylation combined to predict chemotherapy sensitivity were 69.70%, 92.90% and 0.867 (95% CI 0.774–0.960, *P* < 0.001), see Table 4. ROC curve is shown in Fig. 2.

**Table 4:** The ability of CpGs to predict chemotherapy sensitivity in advanced HGSOC

CpGid	AUC	95% CI	P	Cutoff	Sensitivity	Specificity
cg21105318	0.774	0.658–0.890	0.000	42.77	0.970	0.464
cg07896068	0.811	0.703–0.918	0.000	78.21	0.636	0.893
cg18437633	0.785	0.667–0.902	0.000	81.02	0.667	0.786
cg27637873	0.846	0.749–0.944	0.000	77.79	0.818	0.786
cg26782361	0.835	0.735–0.936	0.000	74.21	0.727	0.821
cg16265707	0.814	0.708–0.919	0.000	72.26	0.636	0.857
cg20937934	0.787	0.672–0.902	0.000	63.65	0.879	0.571
cg13270625	0.787	0.672–0.901	0.000	60.16	0.758	0.714
Combined	0.867	0.774–0.960	0.000	68.63	0.697	0.929

### Hypermethylation of CpGs are associated with poor PFS in advanced high-grade serous ovarian cancer (the total group)

**Fig. 3:**
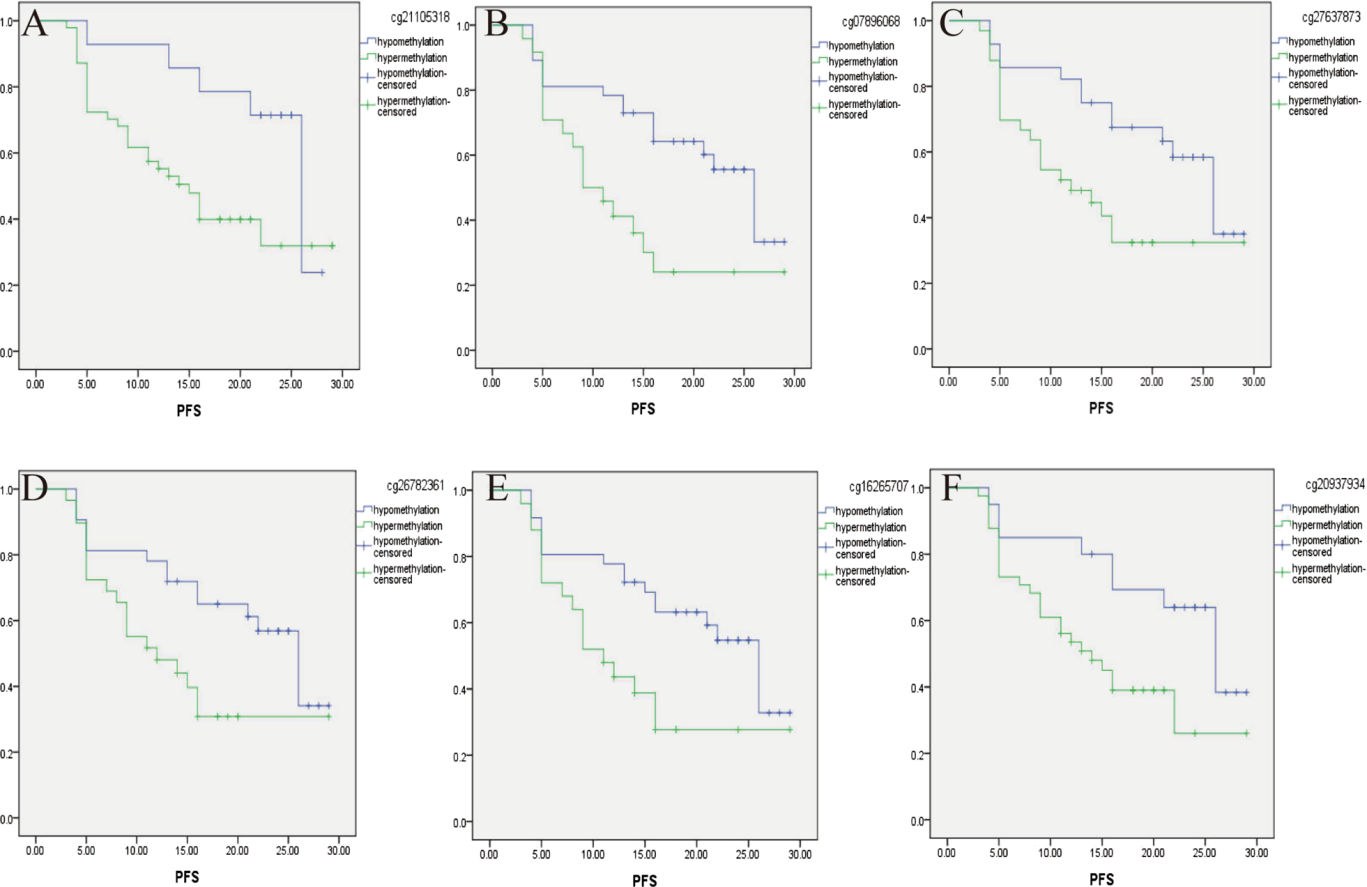
CpGs hypermethylation associated with poor PFS in advanced HGSOC (**a** ITGB6/cg21105318, **b** ITGB6/cg07896068, **c** NCALD/cg27637873, **d** NCALD/cg26782361, **e** NCALD/cg16265707 and **f** LAMA3/cg20937934)

According to the ROC analysis result of the 450 K Infinium Methylation BeadChip, the cutoff values of 8 CpGs (ITGB6:cg21105318, cg07896068, cg18437633; NCALD: cg27637873, cg26782361, cg16265707; LAMA3: cg20937934, cg13270625) methylation were 42.77, 78.21, 81.02, 77.79, 74.21, 72.26, 63.65 and 60.16, respectively, in advanced high-grade serous ovarian cancer (*N* = 61). According to the cutoff value of 8 CpGs methylation, patients were divided into hypomethylation patients and hypermethylation patients. Kaplan–Meier analysis showed that 6 candidate genes (ITGB6:cg21105318, cg07896068; NCALD: cg27637873, cg26782361, cg16265707; LAMA3: cg20937934) hypermethylation can divide patients into high-risk patients and low-risk patients to chemotherapy. Compared with hypomethylation patients, PFS of 6 candidate genes (ITGB6:cg21105318, cg07896068; NCALD: cg27637873, cg26782361, cg16265707; LAMA3: cg20937934) hypermethylation patients was significantly shorter, see Table 5. The survival curve is shown in Fig. 3a–f. There was no significant difference between OS.

**Table 5:** The relationship between CpGs methylation and PFS in advanced HGSOC

CpGid	Advanced HGSOC (the total group)			Advanced HGSOC with complete debulking			Advanced HGSOC with incomplete debulking		
	Mean (months)	95% CI	*P*	Mean (months)	95% CI	*P*	Mean (months)	95% CI	*P*
*cg21105318*									
Hypomethylation	22.98	19.43–26.52	0.020	25.63	22.65–28.60	0.086	20.33	12.63–28.04	0.08
Hypermethylation	16.18	13.13–19.23		18.07	14.00–22.14		12.39	9.00–15.78	
*cg07896068*									
Hypomethylation	20.55	17.44–23.66	0.006	23.50	19.92–27.09	0.004	16.14	11.04–21.25	0.343
Hypermethylation	13.66	9.74–17.57		14.14	8.43–19.86		12.30	8.04–16.56	
*cg27637873*									
Hypomethylation	21.36	18.02–24.70	0.010	23.89	20.20–27.58	0.015	16.67	10.43–22.91	0.437
Hypermethylation	15.18	11.59–18.78		16.27	11.00–21.53		13.00	9.10–16.90	
*cg26782361*									
Hypomethylation	20.66	17.33–23.99	0.019	22.86	18.89–26.83	0.068	17.17	11.55–22.79	0.168
Hypermethylation	15.08	11.35–18.81		17.18	11.84–22.51		11.25	8.04–14.46	
*cg16265707*									
Hypomethylation	20.37	17.21–23.52	0.013	22.89	19.27–26.52	0.022	16.23	10.76–21.71	0.309
Hypermethylation	14.37	10.41–18.33		16.14	10.22–22.06		12.55	8.56–16.53	
*cg20937934*									
Hypomethylation	21.97	18.07–25.87	0.023	24.30	20.15–28.45	0.073	18.00	10.04–25.96	0.212
Hypermethylation	15.63	12.35–18.91		16.80	12.19–21.42		12.88	9.44–16.33

### Relationship between candidate CpGs methylation and PFS in advanced HGSOC patients with different surgical outcomes

**Fig. 4:**
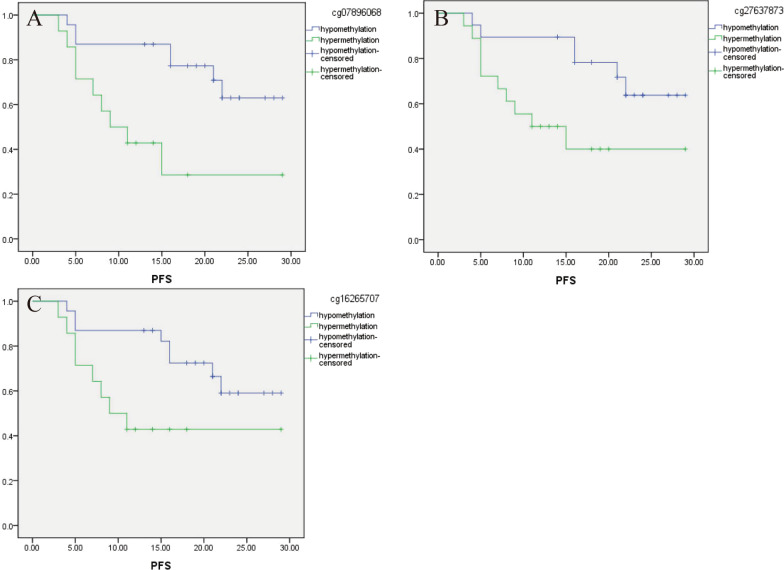
CpGs hypermethylation associated with poor PFS in advanced HGSOC with complete debulking (**a** ITGB6/cg07896068, **b** NCALD/cg27637873 and **c** NCALD/ cg16265707)

Patients with complete debulking have a better progression-free survival. To exclude the effect of the surgical outcome on prognosis, we analyzed the relationship between 6 CpGs and PFS in patients with different surgical outcomes. In advanced high-grade serous ovarian cancer with complete debulking (*N* = 37), PFS of 3 candidate CpGs (ITGB6:cg07896068; NCALD: cg27637873, cg16265707) hypermethylation patients was significantly shorter. The survival curve is shown in Fig. 4a–c. There was no significant difference between PFS in advanced high-grade serous ovarian cancer with incomplete debulking (*N* = 24), see Table 5.

### Identification of candidate CpGs by pyrosequencing in advanced high-grade serous ovarian cancer

**Fig. 5:**
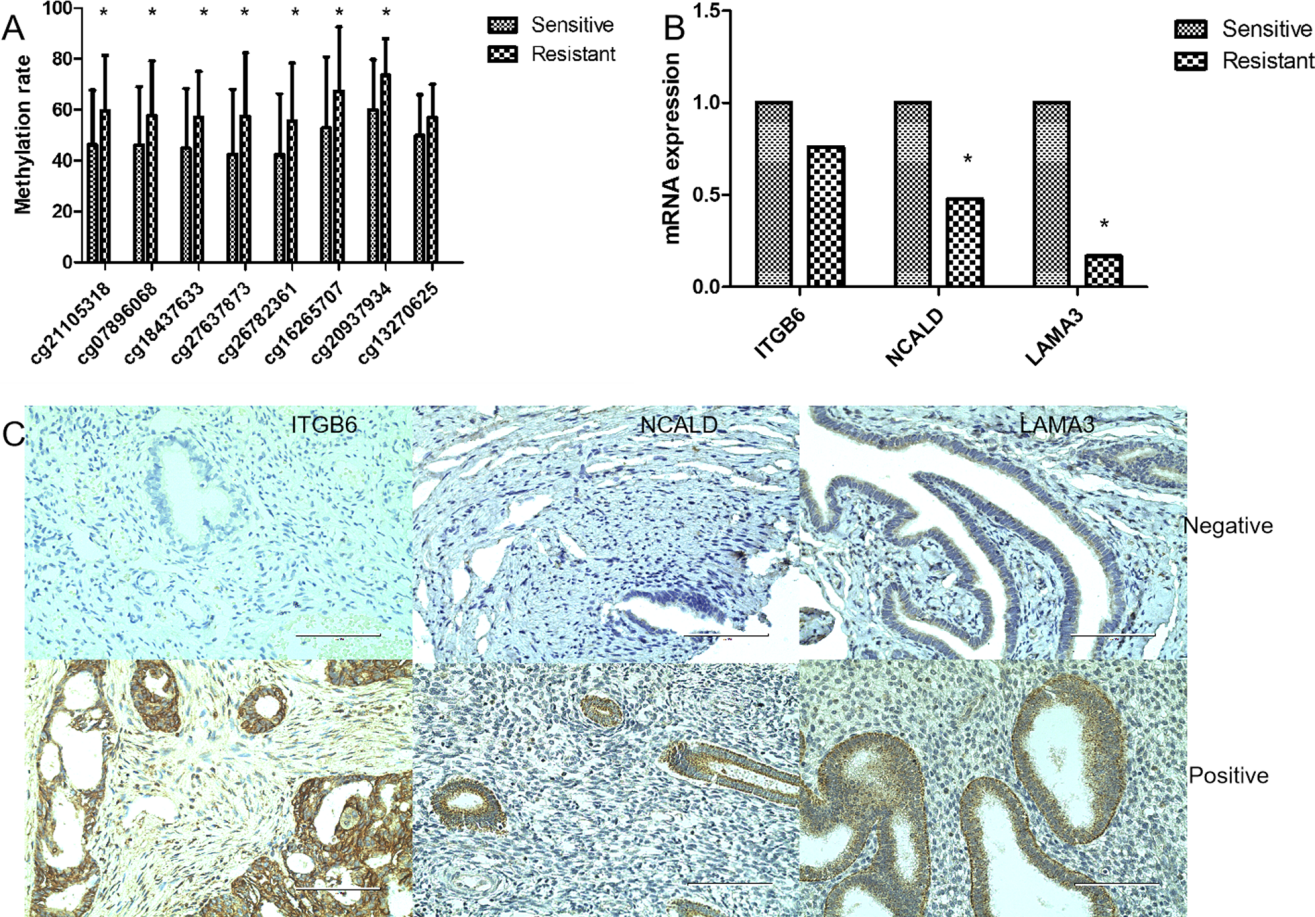
**a** The methylation rate of CpGs in advanced HGSOC (pyrosequencing). **b** Expressions of hypermethylated genes (ITGB6, NCALD and LAMA3) in advanced HGSOC (QRT-PCR). **c** Immunohistochemical stain (400 ×)

Considering that there are certain false positives on the 450 K Infinium Methylation BeadChip, we further verified the 8 candidate CpGs (ITGB6:cg21105318, cg07896068, cg18437633; NCALD: cg27637873, cg26782361, cg16265707; LAMA3: cg20937934, cg13270625) used pyrosequencing in advanced high-grade serous ovarian cancer. In the pyrosequencing results, the methylation of 7 CpGs (ITGB6:cg21105318, cg07896068, cg18437633; NCALD: cg27637873, cg26782361, cg16265707; LAMA3: cg20937934) in the chemoresistant patients were still higher than that in the chemosensitive patients. There was no statistically significant difference in cg13270625 (LAMA3) methylation level between chemoresistant patients and chemosensitive patients. The methylation rate is shown in Fig. 5a.

### Low expression of NCALD and LAMA3 in advanced high-grade serous ovarian cancer

In advanced high-grade serous ovarian cancer, 8 CpGs hypermethylated in chemoresistant patients (ITGB6:cg21105318, cg07896068, cg18437633; NCALD: cg27637873, cg26782361, cg16265707; LAMA3: cg20937934, cg13270625).The mRNA and protein expressions of the corresponding 3 hypermethylated genes (ITGB6, NCALD and LAMA3) in advanced high-grade serous ovarian cancer were detected. QRT-PCR results showed that the expression of NCALD and LAMA3 in chemoresistant patients was lower than those in chemosensitive patients (P = 0.039, P = 0.008). The expression of ITGB6 was not statistically different between chemoresistant patients and chemosensitive patients, see Fig. 5b. In the tissue array composed of 132 advanced high-grade serous ovarian cancer patient samples, immunohistochemistry also showed that the expression of NCALD and LAMA3 in chemoresistant patients was lower than that of chemosensitive patients (57.89% VS 39.58%, *P* = 0.048; 54.67% VS 42.55%, *P* = 0.041). The expression of ITGB6 was not statistically different between chemoresistant patients and chemosensitive patients (55.56% VS 52.08%, P = 0.71), which was consistent with mRNA expression results. The staining chart is shown in Fig. 5c.

### NCALD and LAMA3 expression are negatively correlated with methylation in advanced high-grade serous ovarian cancer

**Fig. 6:**
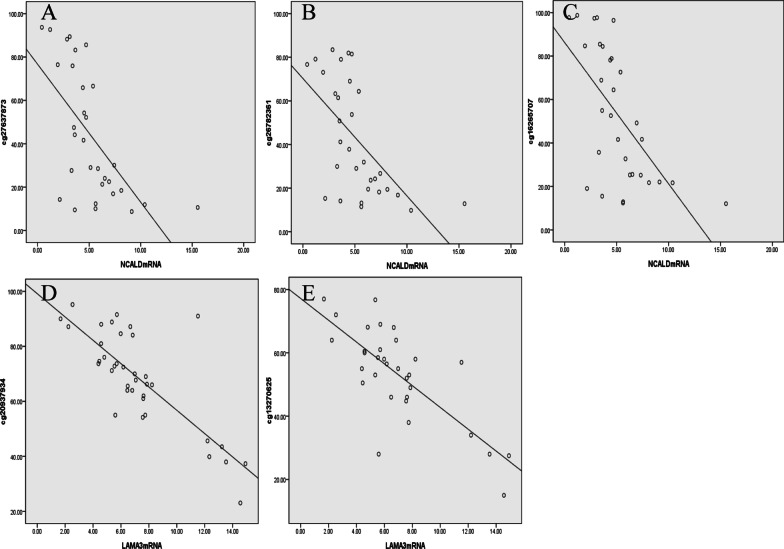
The relationship between NCALD and LAMA3 methylation and mRNA expression in advanced HGSOC (**a** cg27637873 methylation and NCALD expression. **b** cg26782361 methylation and NCALD expression. **c** cg16265707 methylation and NCALD expression. **d** cg20937934 methylation and LAMA3 expression. **e** cg13270625 methylation and LAMA3 expression)

In advanced high-grade serous ovarian cancer, NCALD and LAMA3 have hypermethylation and low expression in chemoresistant patients. Spearman analysis showed that there was a negative correlation between the methylation of NCALD (cg27637873, cg26782361, cg16265707) and LAMA3 (cg20937934, cg13270625) and their mRNA expression. The correlation coefficients between the methylation of NCALD (cg27637873, cg26782361, cg16265707) and mRNA expression were -0.669, -0.636 and -0.657, respectively (*P* < 0.05). The correlation coefficients between the methylation of LAMA3 (cg20937934, cg13270625) and mRNA expression were − 0.726 and − 0.649, respectively (*P* < 0.05). See Fig. 6a–e. The hypermethylation of NCALD and LAMA3 in promoters may be the cause of its downregulation in chemoresistance ovarian cancer patients.

### Correlation between candidate genes in advanced high-grade serous ovarian cancer

The regulatory mechanism of DNA methylation in the development of chemotherapy resistance in ovarian cancer is complex and diverse. A variety of methylation genes interact with each other, which together leads to chemotherapy resistance in ovarian cancer. We found that among the 8 candidate CpGs (ITGB6:cg21105318, cg07896068, cg18437633; NCALD: cg27637873, cg26782361, cg16265707; LAMA3: cg20937934, cg13270625) in advanced high-grade serous ovarian cancer, the methylation level was highly positively correlated between any two CpGs, and the CpG correlation coefficient of the same gene was higher. See Additional file 1: Table S5. Further analysis of gene expression correlation revealed that NCALD protein expression was positively correlated with LAMA3 and ITGB6 protein expression. See Additional file 1: Table S6.

## Discussion

Clinically, there are limited ways to treat chemotherapy resistance in ovarian cancer. However, demethylation drugs have been shown to resensitize ovarian cancer patients to platinum chemotherapy. It can be seen that abnormal methylation is a key factor in the formation of chemotherapy resistance in ovarian cancer. In this study, we used 450 K Infinium Methylation BeadChip to detect 7263 different CpGs in ovarian cancer chemoresistant and chemosensitive patients. We systematically searched the literature in the PubMed database and obtained 54 methylated genes related to chemotherapy resistance in ovarian cancer. Among them, 22 methylation genes related to ovarian resistance reported in the literature are enriched in our Methylation BeadChip results. The difference of BRCA1, CD133, ASS1, ABCG2, TGFBI, RGS10, UCHL1, CLDN4, HOXA10, DOK2, AGR2 and OXCT1 gene in our 450 K Infinium Methylation BeadChip is consistent with that reported in the literature. BRCA1 is a drug-related gene in ovarian cancer that has received attention. It has been reported to be involved in many cellular processes, including DNA repair and recombination, cell cycle regulation, chromatin remodeling and ubiquitination [66]. Ignatov's study showed that PFS was significantly prolonged in patients with BRCA1 promoter methylation in recurrent ovarian cancer (18.5 months vs 12.8 months, *P* = 0.008) [67]. Stefansson confirmed that BRCA1 hypermethylation increased platinum sensitivity in ovarian cancer cell lines, xenograft tumors and clinical samples [68]. However, some studies have reported that BRCA1 methylation reduces the sensitivity of the tumors to platinum drugs. For example, Wang’s study showed that with the progress of ovarian cancer, the methylation rate of the BRCA1 promoter increased significantly. Hypermethylation of the BRCA1 gene can lead to the loss of BRCA1 protein and RNA, which makes the disease of these patients develop faster and shorten the survival time than those without BRCA1 methylation [69]. Patch also proposed that, compared with BRCA1/2 gene mutation and expression downregulation, BRCA1 promoter methylation is related to platinum resistance [70]. In our Methylation BeadChip results, BRCA1 is hypomethylated in resistant patients, which is consistent with the results of Stefansson and Ignatov. The relationship between BRCA1 methylation and drug resistance in ovarian cancer still needs further verification. In our Methylation BeadChip results, combined with the region of CpGs, KEGG, GO and prognosis analysis, 9 new candidate CpGs methylation were selected to be associated with drug resistance of epithelial ovarian cancer. Ovarian cancer is not a single disease and can be subdivided into at least five different histological subtypes that have different identifiable risk factors, cells of origin, molecular compositions, clinical features and treatments [71]. High-grade serous ovarian cancer (HGSOC) is the most common ovarian cancer subtype. The vast majority of HGSOC cases are diagnosed at advanced stages (FIGO stage III and IV) with 5-year survival rates of approximately 39% and 17%, respectively [72]. In advanced high-grade serous ovarian cancer, 8 CpGs remained hypermethylated in chemoresistant patients.

Ovarian cancer is a highly heterogeneous disease characterized by multiple histological subtypes. Molecular diversity has been shown to occur within specific histological subtypes of ovarian cancer, between different tumors of an individual patient, as well as within individual tumors. Therefore, there are no clinically validated markers for chemotherapy sensitivity in ovarian cancer. Genome-wide DNA methylation detection helps to understand the complex characteristics of DNA methylation mutations. At present, studies on genome-wide DNA methylation of chemotherapy resistance in ovarian cancer have been largely limited to the level of cell and animal xenografts [52, 73]. Very few studies have examined the genome-wide DNA methylation characteristics of chemoresistant patients. Tomar [12] detected the genome-wide methylation of 8 chemosensitive ovarian cancer patients and 10 chemoresistant ovarian cancer patients. It was found that there were 45 differentially methylated and expressed genes between patients with two chemotherapy outcomes; In the same patient, pyrosequencing confirmed 9 different methylation genes. In the verification set, there are 4 candidate genes (FZD10, FAM83A, MYO18B and MKX) that have at least one CpG site with significant differences between patients with two chemotherapy outcomes. Compared with previous studies on the genome-wide methylation of ovarian cancer chemotherapy resistance, our study has several advantages. First, compared with cell or animal xenografts models, our genome-wide methylation and expression data were obtained from clinical surgical specimens, with long-term follow-up and chemotherapy outcomes. Second, genome-wide methylation was detected in 53 chemosensitive patients and 55 chemoresistant patients. The sample size was significantly increased. Third, unlike previous studies that only focused on screening a large number of markers, we also compared the diagnostic efficacy of abnormal methylation markers to predict chemoresistance. This provides a direction for the early identification of chemoresistant ovarian cancer patients in clinical. We identified 3 new methylation genes (ITGB6, NCALD and LAMA3) with different chemotherapy outcomes in advanced high-grade serous ovarian cancer. Patients with ITGB6, NCALD and LAMA3 hypermethylation have a poor prognosis. The methylation of the NCALD and LAMA3 is negatively correlated with their mRNA expression.

NCALD (neurocalcin delta) is a member of the neuron calcium sensor family, which is involved in the calcium signal pathway and G protein coupled receptor signal pathway. A bioinformatics study in 2020 showed that NCALD expression is regulated by DNA methylation and microRNAs. TCGA data found that the expression of NCALD in platinum-resistant patients was lower than that in platinum-sensitive patients. Patients with low NCALD expression have poor overall survival (OS) and progression-free survival (PFS) [74]. Our findings are consistent with this report. Epigenetic inactivation of NCALD may be one of the key factors leading to chemoresistance in ovarian cancer patients. LAMA3 (laminin, alpha 3) is an important component of the cell basement membrane and plays an important role in the process of cell adhesion, cell migration and embryo differentiation. As an epigenetic inactivation gene, LAMA3 has been reported in various cancer development and chemoresistance studies. However, there is only one study of LAMA3 in ovarian cancer. Tang [75] found that the methylation of LAMA3 in ovarian cancer tissues was higher than that in adjacent tissues and normal tissues. The expression of LAMA3 in ovarian cancer tissues was lower than that in adjacent tissues and normal tissues. The relationship between LAMA3 and ovarian cancer chemoresistance has not been reported. Our study found for the first time that LAMA3 was abnormally hypermethylated and silenced in chemoresistant ovarian cancer patients, which may be a target gene of epigenetic therapy. Although NCALD and LAMA3 are genes that show both methylation and expression changes, the methylation of the ITGB6 gene may also play a role in the chemoresistance of ovarian cancer.

Because the mechanism of chemoresistance in ovarian cancer is complex and diverse, it is very difficult to predict the chemotherapy outcome of ovarian cancer. Abnormal methylation can stratify ovarian cancer patients according to the chemotherapy outcome. In advanced high-grade serous ovarian cancer, the sensitivity, specificity and AUC of 8 CpGs methylation to predict chemotherapy sensitivity were 63.60–97.00%, 46.40–89.30% and 0.774–0.846. PFS of 6 candidate genes hypermethylation patients was significantly shorter. Residual lesions after primary surgery are another important prognostic factor in patients with advanced ovarian cancer. Patients with complete debulking have a better progression-free survival. Incomplete debulking cannot improve the prognosis, and it may even lead to more perioperative morbidity. Therefore, we analyzed the relationship between CpGs methylation and PFS in patients who with complete debulking or incomplete debulking, respectively. In advanced high-grade serous ovarian cancer with complete debulking, PFS of 3 candidate CpGs (ITGB6:cg07896068; NCALD:cg27637873, cg16265707) hypermethylation patients was significantly shorter. In advanced high-grade serous ovarian cancer with incomplete debulking, there was no significant difference between candidate CpGs hypermethylation and PFS. It is suggested that the methylation of 3 CpGs is more valuable in predicting the prognosis of patients with complete debulking.

Like Zhang's research, we also observed ‘batch effect’ in pyrosequencing [76]. Abnormal DNA methylation was found to be associated not only with disease [77, 78], but also with patient age, FIGO stage and histological type. This suggests that we need to stratify potential clinical factors when analyzing methylation related to chemotherapy resistance. Interestingly, we found that among the 8 CpGs in advanced high-grade serous ovarian cancer, the methylation level was highly positively correlated between any two CpGs, and the CpG correlation coefficient of the same gene was higher. Corresponding 3 hypermethylated genes, NCALD protein expression was positively correlated with LAMA3 and ITGB6 protein expression. This shows that NCALD, LAMA3 and ITGB6 may influence each other and participate in the chemotherapy resistance of ovarian cancer together.

There are several shortcomings in our study. First, there are challenges in the clinical collection of matching tumor samples before and after chemotherapy. This study is lateral study. The cancer samples selected are the initial surgical samples. However, our data can still show that the acquisition of these gene methylations is the potential molecular characteristic to obtain chemotherapy resistance and poor prognosis. Second, it is particularly important to predict the response to chemotherapy in ovarian cancer patients with incomplete debulking. Our data reveal that candidate CpGs hypermethylation is associated with worse PFS only in advanced HGSOC patients with complete debulking, but not in advanced HGSOC patients with incomplete debulking. It is suggested that the prognosis prediction of advanced HGSOC patients with incomplete debulking is more complicated. In the future, we will need to combine other biomarkers (such as BRCA1/2 mutational status) or further optimize the model for these specific populations. Third, the biological mechanism of candidate markers such as NCALD and LAMA3 is unclear. We speculate that there is a certain connection between the differentially methylated genes and the combined effect that leads to chemotherapy resistance. This functional mechanism needs to be further studied through confirmatory studies.

## Conclusions

In summary, our study shows extensive methylation differences in chemosensitive and chemoresistant ovarian cancer patients. In advanced high-grade serous ovarian cancer, ITGB6, NCALD and LAMA3 hypermethylation indicate chemotherapy resistance and poor prognosis. Important new findings include the identification of two new key genes, NCALD and LAMA3, which may drive the acquired resistance of ovarian cancer. For the first time, the role of DNA methylation in regulating the function of NCALD and LAMA3 genes in advanced high-grade serous ovarian cancer was pointed out. This not only enriches the new gene pool for chemoresistance mechanisms of ovarian cancer, but also provides direction for finding stratified markers of epigenetic therapy.

## Supplementary Information

**Additional file 1**.** Table S1**. Amplification primers and sequencing primers for Pyrosequencing.** Table S2**. Primers sequence for QRT-PCR.** Table S3**. Pathway analysis of different methylation sites between sensitive and resistant patients in KEGG (epithelial ovarian cancer).** Table S4**. Go analysis of different methylation sites in sensitive and resistant patients (epithelial ovarian cancer).** Table S5**. Correlation between candidate cpgs methylation (advanced HGSOC).** Table S6**. Correlation between candidate genes expression (advanced HGSOC).

## Data Availability

Not applicable.

## Abbreviations

CpGs: Cytosine-phosphate-guanine site
GO: Geneontology databases
KEGG: Kyoto Encyclopedia of Genes and Genomes
QRT-PCR: Quantitative real-time PCR
IHC: Immunohistochemistry
ROC: Receiver operating characteristic
ITGB6: Integrin beta 6
NCALD: Neurocalcin delta
LAMA3: Laminin alpha 3
AUC: Area under curve
OS: Overall survival
PFS: Progression-free survival
PCR: Polymerase chain reaction
PIK3R3: Phosphoinositide-3-kinase, regulatory subunit 3 (gamma)
FIGO: The International Federation of Gynecology and Obstetrics
